# A Companion Diagnostic With Significant Clinical Impact in Treatment of Breast and Gastric Cancer

**DOI:** 10.3389/fonc.2021.676939

**Published:** 2021-07-23

**Authors:** Jan Trøst Jørgensen, Henrik Winther, Jon Askaa, Lena Andresen, Dana Olsen, Jens Mollerup

**Affiliations:** ^1^ Dx-Rx Institute, Fredensborg, Denmark; ^2^ Biovica International AB, Uppsala, Sweden; ^3^ Independent Researcher, Frederiksberg, Denmark; ^4^ Agilent Technologies Denmark ApS, Glostrup, Denmark

**Keywords:** HercepTest, companion diagnostics, drug-diagnostic codevelopment, breast cancer, gastric cancer, trastuzumab, pertuzumab, ado-trastuzumab emtansine

## Abstract

The development of trastuzumab (Herceptin^®^) was one of the most significant cancer drug development projects of the 20th century. Not only was it a scientific and medical achievement but it also paved the way for the drug-diagnostic codevelopment model, where a predictive biomarker assay is developed in parallel to the drug. One of the challenges in the development of trastuzumab was to select the right patient population likely to respond and here, it was critical to have access to an accurate, robust and reliable assay for detection of HER2 overexpression in tumors. In the clinical development of trastuzumab, a clinical trial assay (CTA), developed by Genentech, was used for selection of HER2 positive patients. However, during the phase III trial with trastuzumab, a new optimized IHC assay, HercepTest™ was designed and developed by Dako. In the final stage of its development, a comparative study with the CTA was conducted in order to show concordance between the two assays. In September 1998, the Food and Drug Administration (FDA) simultaneously granted approval to trastuzumab and HercepTest™. The assay has been used for patient selection in a number of significant breast cancer clinical trials such as the HERA, CLEOPATRA, EMILIA and more. In these trials, HercepTest™ demonstrated its clinical utility in the neoadjuvant, adjuvant, and metastatic setting as well as in relation to different types of HER2 targeted therapies. Likewise, the assay was used for selection of HER2 positive gastric cancer patients in the important ToGA trail. HercepTest™ was the first companion diagnostic ever approved by the FDA, and more than 20 years of use has documented its clinical impact.

## Introduction

In 2019, when Dennis Slamon, Axel Ullrich, and Michael Shepard received the Lasker-DeBakey Clinical Medical Research Award, the *New England Journal of Medicine* published an article by the former American Society of Clinical Oncology (ASCO) president Daniel Hayes, entitled ‘HER2 and Breast Cancer - A Phenomenal Success Story’ (1). The development of trastuzumab (Herceptin^®^, Roche/Genentech) was definitely a phenomenal success story. Trastuzumab was the first monoclonal antibody targeted towards an oncoprotein and it significantly changed the approach to cancer drug development and the treatment of the approximately 20% of women with HER2 positive breast cancer. Not only was this a scientific and medical achievement, but it also paved the way for the drug-diagnostic codevelopment model where a predictive biomarker assay is used to select the patients most likely to respond (1–3). More importantly, for women with HER2-positive breast cancer, treatment with trastuzumab meant a substantial improvement in survival and a sustained reduction in cancer recurrence (4–6).

Dennis Slamon, Axel Ullrich, and others published an article in *Science* in 1987, where they described the link between amplification of the *HER2* gene and a poor prognosis of women with breast cancer (7). They concluded that the *HER2* gene product functioned as a growth factor receptor that played a role in the pathogenesis of breast cancer and that the development of a specific receptor antagonist could have important therapeutic implications. The germ was laid for one of the most significant cancer drug development projects of the 20th century. The antagonist mentioned in their *Science* paper, later became the monoclonal antibody trastuzumab and when Genentech entered clinical development, an immunohistochemistry (IHC) assay that could detect HER2 protein expression levels in tumor tissue, called the clinical trial assay (CTA), was developed. In the early clinical development of trastuzumab, the CTA was used for patient selection and the different clinical trials demonstrated a link between HER2 overexpression and the efficacy of trastuzumab (8–10). By using the CTA for patient stratification, Genentech formed the basis for the prospective enrichment clinical trial design, which is well-established in today’s cancer drug development. In September 1998, the Food and Drug Administration (FDA), through a new coordinated procedure, simultaneously granted approval to trastuzumab and a new optimized HER2 IHC assay, named HercepTest™ (Dako). This simultaneous approval made sense, as the assay is an important treatment decision tool that needs to be available at the same time as the drug (11).

One of the challenges in the development of trastuzumab was to identify and hence select the right patient population likely to respond and it was critical to have access to an accurate and reliable diagnostic assay for detection of HER2 protein overexpression in the tumor (12). Following the approval of trastuzumab for treatment of metastatic breast cancer, another former ASCO president, Gabriel Hortobagyi, stated in an article in *Seminars in Oncology*: ‘If an assay did not exist to identify the patient population likely to respond to therapy, trastuzumab might have been discarded during development because of insufficient activity in an unselected patient population.’ (13). This claim was further substantiated by Richard Simon and Aboubakar Maitournam, when they published their alternative sample size calculations in *Clinical Cancer Research* (14, 15). These calculations were based on the outcome data from the phase III trial with trastuzumab in metastatic breast cancer, where 469 patients were randomized to chemotherapy or chemotherapy plus trastuzumab (10). Their calculations showed that if an all-comers trial design had been used, without testing for HER2 overexpression, the number of patients needed to be enrolled in the phase III trial would have been more than 8,000, in order to demonstrate the same statistically significant difference between the two arms in the trial (14, 15). This would have been 17 times more patients than in the actual phase III trial and showed how crucial the CTA was for the development of trastuzumab.

Over the years, the development of trastuzumab has been widely described but very little has been published about the first companion diagnostic assay, HercepTest™, for detection of HER2 tumor expression. This review will focus on the development of HercepTest™ and especially its clinical utility in relation to treatment of patients with breast cancer and gastric cancer.

## Breast Cancer

### Clinical Trials Assay (CTA)

Throughout all phases of the clinical development of trastuzumab, an IHC assay called the CTA developed by Genentech was used to detect HER2 tumor overexpression in possible eligible study patients with breast cancer. This assay utilized two different mouse monoclonal antibodies, the parent murine antibody to trastuzumab, clone 4D5, and anti HER2, clone CB11. The majority of tumor specimens in the different clinical trials with trastuzumab were tested using the 4D5 antibody. The CTA was a technically challenging IHC assay with more than 35 individual process steps, and it required four or more tissue sections per patient for completion (11).

In the early phase clinical trials with trastuzumab, different patient selection criteria for HER2 overexpression were used (8). However, with the conduct of the pivotal phase II trial in patients with metastatic breast cancer, where trastuzumab was given as monotherapy, the scoring criteria for the HER2 expression more or less found its final form. These scoring criteria were subsequently used throughout the clinical development program (16). In this phase II trial, the IHC staining was performed with either the 4D5 or CB11 antibody, and the HER2 tumor expression was scored as 0, 1+, 2+, or 3+ using standardized scoring criteria, as shown in **Table 1** (17). Patients with weak (HER2 IHC2+) or complete (HER2 IHC3+) cell membrane staining of > 10% of the tumor cells were eligible for treatment with trastuzumab and were enrolled in the clinical trial. Among the 222 patients with metastatic breast cancer, the objective response rate (ORR) was 15%. Patients with HER2 IHC3+ tumors showed a somewhat higher ORR than those with HER2 IHC2+ tumors, 18% *versus* 6% (17).

**Table 1:** The FDA approved scoring criteria for HercepTest™ in breast cancer (17).

Score	HER2 protein overexpression assessment	Staining pattern
0	Negative	No staining is observed or membrane staining is observed in less than 10% of the tumor cells
1+	Negative	A faint/barely perceptible membrane staining is detected in more than 10% of the tumor cells. The cells are only stained in part of their membrane
2+	Weakly positive	A weak to moderate complete membrane staining is observed in more than 10% of the tumor cells
3+	Strongly positive	A strong complete membrane staining is observed in more than 10% of the tumor cells

The final phase III trial with trastuzumab in metastatic breast cancer also used the CTA for enrollment and here, 469 patients with either a HER2 IHC2+ or a HER2 IHC3+ score were randomized to chemotherapy (doxorubicin/epirubicin plus cyclophosphamide or paclitaxel alone) or chemotherapy plus trastuzumab (10). The study showed that addition of trastuzumab to chemotherapy was associated with a higher ORR (50% *versus* 32%; P<0.001), a longer time to disease progression (median, 7.4 *versus* 4.6 months; P<0.001), and a 20% reduction in the risk of death. Again, it was shown that patients with a score of HER2 IHC3+ benefitted more from treatment with trastuzumab than those with a score of HER2 IHC2+. During the clinical development of trastuzumab, more than 7,000 tumor samples were tested in a single centralized laboratory using the CTA (11).

### HercepTest™ for Breast Cancer

Due to the complexity of the CTA, it was not considered robust enough to be developed for a broader use in the general pathology laboratories. In order to standardize the assay and make it more robust and reliable for use in routine diagnostic laboratories, Genentech began a cooperation with the Danish diagnostic company Dako in 1996 (11). During the initial development of the new HER2 IHC assay, selection of the optimal primary antibody, epitope retrieval and staining procedure took place. Different primary antibodies were assessed, which included the two mouse monoclonal antibodies to HER2, clone 4D5 and clone CB11 as well as an affinity-purified, polyclonal rabbit antibody. In order to enhance the performance of the assay, a two-step immunohistochemical staining procedure using the EnVision visualization system (Dako) was used. The development work with the different prototype assays showed that the Dako polyclonal antibody A0485 in combination with the EnVision visualization system was superior with regard to immunohistochemical sensitivity and specificity compared to assays based on the two mouse monoclonal antibodies utilized in the CTA. Based on the prototype testing, the polyclonal rabbit antibody was chosen as the primary antibody for the new assay, later named HercepTest™ (11).

Several concordance studies were performed with the HercepTest™, which also included a comparison to *HER2* fluorescence *in situ* hybridization (FISH) using the PathVysion^®^ *HER2* DNA Probe Kit (Abbott Diagnostics). In FISH, the *HER2* gene copy number in relevant tumor cells was determined and reported relative to the copy number of a centromere 17 (CEN17) reference probe determined in the same cells. Using *HER2*/CEN17 ≥ 2.0 as cut-off value for *HER2* amplification by FISH, the study showed a binary concordance of 86% (11). Other studies have subsequently estimated the concordance between *HER2* FISH and HercepTest™ to be around 95% (18, 19). A final concordance study between the CTA and HercepTest™ was performed including 548 breast cancer specimens. These specimens were supplied from the Cooperative Breast Cancer Tissue Resource following approval of the concordance study by the National Cancer Institute. Approximately 50% of these specimens were HER2 positive as determined by the CTA, with a more or less equal distribution of HER2 IHC2+ and IHC3+ cases. The binary concordance between the two assays was 79% (95% CI; 76% - 82%), which was above the prespecified acceptance criteria of 75% (11).

**Figure 1:**
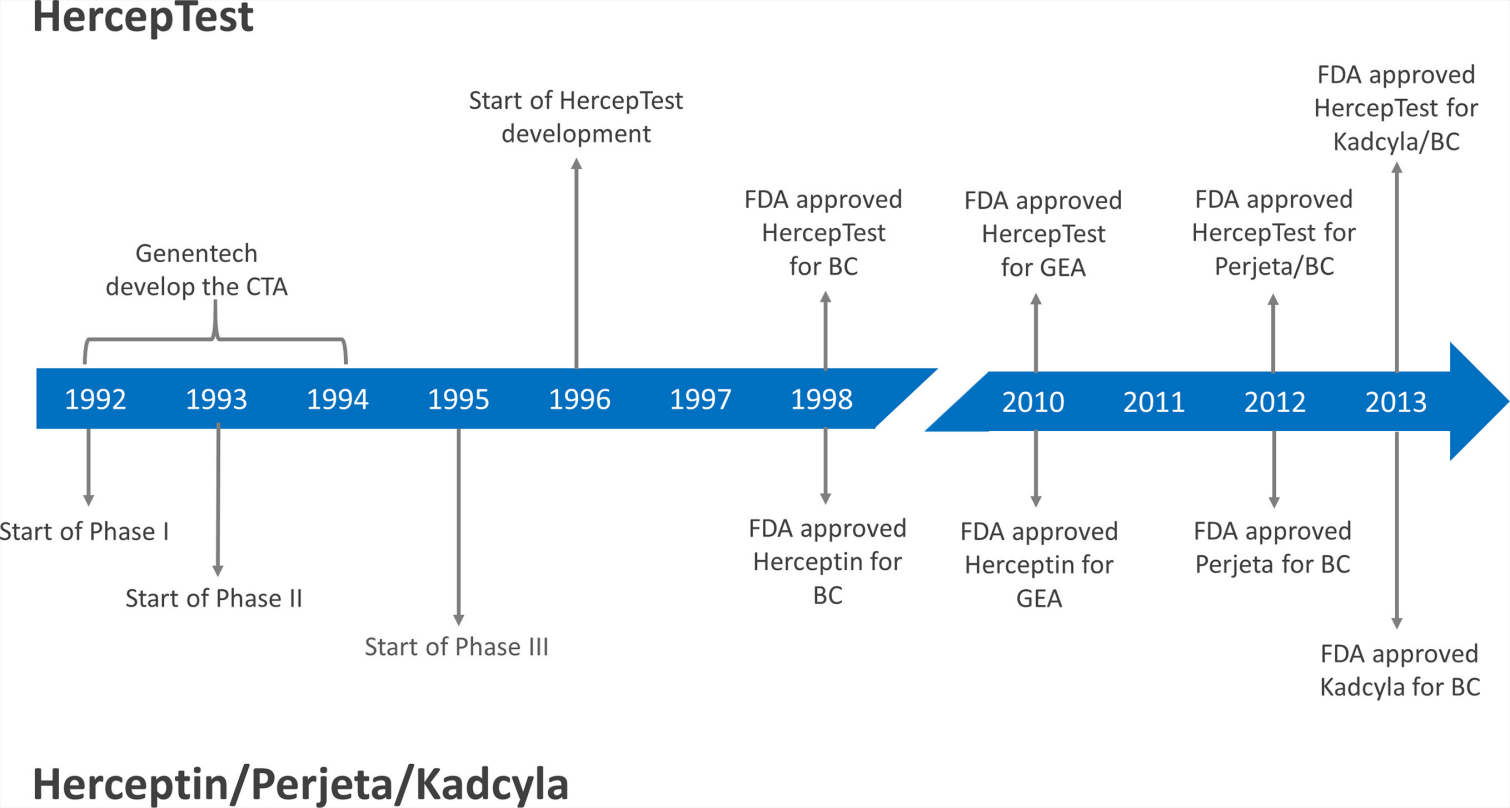
Milestones for the development of HercepTest™ and different HER2 targeted therapies. CTA, Clinical Trial Assay; FDA, Food and Drug Administration; BC, Breast Cancer; GEA, Gastroesophageal junction adenocarcinoma; Herceptin^®^, trastuzumab; Perjeta^®^, pertuzumab; Kadcyla^®^, ado-trastuzumab emtansine/TDM1.

Following positive evaluations at an Oncologic Drugs Advisory Committee Meeting and a Device Panel Meeting, the FDA approved both trastuzumab and HercepTest™ in September 1998, as shown in **Figure 1** (11). It was important to have a robust and reliable assay available that was easy to distribute to routine diagnostic laboratories at the same time as trastuzumab was approved for clinical use. However, looking retrospectively at the approval of HercepTest™, there are some questionable aspects from today’s point of view. There were no prospective or retrospective clinical outcome data following treatments with trastuzumab that could be linked directly to HercepTest™. This deficiency was also noted in relation to the intended use for HercepTest™, where FDA stated: ‘The actual correlation of the HercepTest™ to Herceptin^®^ clinical outcome has not been established’ (17). However, what was missing with regard to data on the clinical utility of HercepTest™ at the time of approval came in abundance later on.

**Figure 2:**
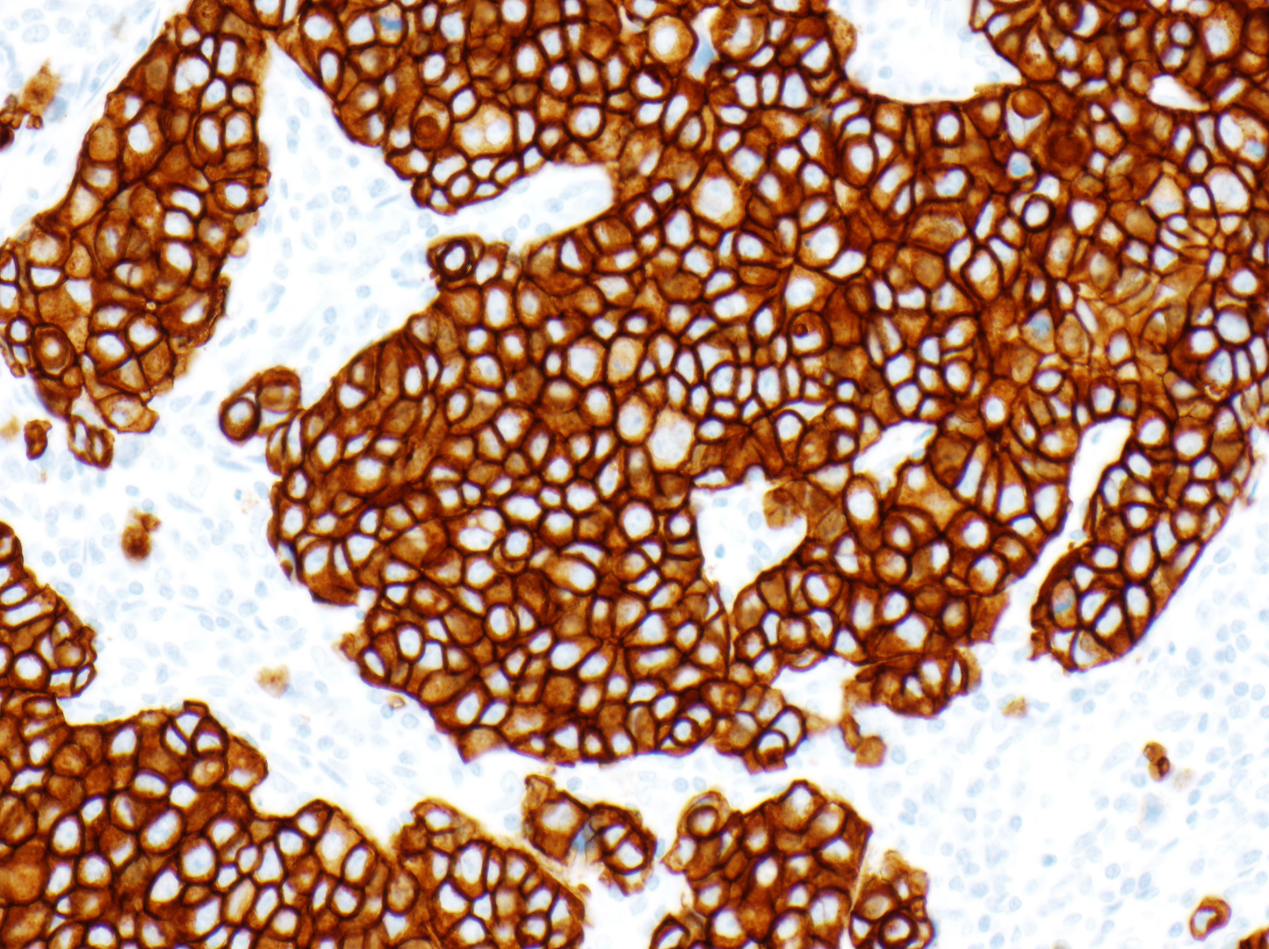
Breast carcinoma stained with HercepTest™, IHC3+ score.

The intended use granted by FDA for HercepTest™ in 1998 stated that it is a semiquantitative IHC assay to determine HER2 overexpression in breast cancer tissues, routinely processed for histological evaluation. Furthermore, in relation to the predictive properties, it is stated that HercepTest™ is indicated as an aid in the assessment of patients for whom Herceptin^®^ (trastuzumab) treatment is being considered (11). Together with the approval of HercepTest™ the FDA also approved the scoring criteria, as shown in **Table 1**. Based on the tumor cell membrane staining pattern, the level of HER2 expression at the time of biopsy is categorized into the four categories of: IHC0 (negative), IHC1+ (negative), IHC2+ (weakly positive), and IHC3+ (strongly positive). **Figure 2** shows a breast carcinoma tissue section with a strong expression of HER2 (IHC3+) following staining with HercepTest™.

### Clinical Data in Breast Cancer

The FDA approval of HercepTest™ was based on analytical data and concordance to the CTA. Since the patients in all the trastuzumab clinical trials were selected using the CTA, a study to compare this assay to HercepTest™ was conducted. The study was, as previously described, performed on independent samples, not originating from the trastuzumab clinical trials. Thus, the actual correlation of HercepTest™ to patient outcome following treatment with trastuzumab was not initially established. The clinical utility of the assay was documented in the years following the approval, where data from a large number of prospective clinical trials were published. HercepTest™ has been used for patient selection in a number of significant breast cancer trials such as the HERA, CLEOPATRA, EMILIA, and more (4, 20–35). In these trials, HercepTest™ has demonstrated its clinical utility in the neoadjuvant, adjuvant, and metastatic setting as well as in relation to different types of HER2 targeted therapies.

One of the more significant clinical trials conducted with HER2 inhibitors in the past decade is CLEOPATRA (29). Here, 808 HER2-positive patients with metastatic breast cancer were randomized to receive placebo plus trastuzumab plus docetaxel or pertuzumab plus trastuzumab plus docetaxel as first-line treatment. Pertuzumab (Perjeta^®^, Roche/Genentech) is a humanized monoclonal antibody that binds to subdomain II of the extracellular part of the HER2 receptor, in contrast to trastuzumab, which binds to subdomain IV. In the trial, HER2-positivity was defined as IHC3+ or amplification by FISH (*HER2*/CEN17 ≥ 2.0). The patients were selected based on results following testing with HercepTest™ and/or *HER2* FISH pharmDx (Dako). The study showed a significant higher median progression-free survival (PFS) for the pertuzumab group compared to the control group, 18.5 months *versus* 12.4 months (HR 0.62; 95% CI, 0.51 - 0.75; P<0.001). Likewise, a statistical significantly higher ORR was observed for the pertuzumab group compared to the control group, 80.2% *versus* 69.3% (P<0.001). Based on data from the CLEOPATRA trial, a concomitant FDA approval of pertuzumab and HercepTest™ was granted in 2012 (see **Figure 1**). The intended use statement for HercepTest™ was expanded to include the use of the assay as an aid in the assessment of breast cancer patients for whom treatment with pertuzumab is being considered (17). Another significant clinical trial, where HercepTest™ was used, is EMILIA (31). In this trial, 990 metastatic breast cancer patients previously treated with trastuzumab and a taxane were randomized to ado-trastuzumab emtansine (T-DM1) or lapatinib plus capecitabine. Ado-trastuzumab emtansine (Kadcyla^®^, Roche/Genentech) is an antibody-drug conjugate consisting of trastuzumab covalently linked to the cytotoxic agent DM1. The HER2 selection criteria were similar to those of CLEOPATRA and the trial showed that ado-trastuzumab emtansine was superior to lapatinib plus capecitabine with respect to median PFS, median overall survival (OS), and ORR. Based on data from the EMILIA trial, a concomitant FDA approval of ado-trastuzumab emtansine and HercepTest™ was granted in 2013 (see **Figure 1**). Again, the intended use statement for HercepTest™ was expanded to include ado-trastuzumab emtansine (17).

For some patients tested positive with a HER2 IHC or a *HER2 in situ* hybridization (ISH) assay, treatment with trastuzumab or other HER2 inhibitors does not always result in a positive clinical outcome and in these cases the test result must be regarded as ‘false positive’, which likely is due to involvement of other molecular pathways than HER2 in the tumor progression. For the CLEOPATRA trial and the use of HercepTest™, the group of patients who were tested positive and received treatment with pertuzumab plus trastuzumab plus docetaxel had an ORR of 80.2% (29). In the CLEOPATRA trial where molecular enrichment was used, ORR is identical to the positive predictive value of the assay.

In the CLEOPATRA trial, a wide range of different biomarkers related to the HER2 pathway were explored for their predictive value, such as *PIK3CA*, PTEN, pAKT, and more (33). Based on the results from this comprehensive analysis, it was concluded that HER2 protein overexpression or *HER2* gene amplification are the only markers suited for patient selection for the trastuzumab plus pertuzumab–based regimen in patients with HER2-positive metastatic breast cancer. This conclusion again underlines the importance of HER2 protein overexpression or *HER2* gene amplification as predictive biomarkers for HER2 targeted therapies in patients with breast cancer.

### HercepTest™ Cut-Off Value

Both during the clinical development of trastuzumab and a few years after its approval, patients with both HER2 IHC2+ and IHC3+ tumors were offered treatment and enrolled in different clinical trials (10, 16, 21–23). However, several trials had showed that trastuzumab was more effective in patients with HER2 IHC3+ than HER2 IHC2+ tumors and the treatment criteria were gradually revised (10, 16, 21). When the different adjuvant trials with trastuzumab were set up, the selection criteria changed. In the adjuvant HERA trial, HER2 positivity was defined as either HER2 IHC3+ or *HER2* amplification using FISH (*HER2*/CEN17 ≥ 2.0). Furthermore, for patients with HER2 IHC2+ tumors, a reflex test using FISH was performed and if the tumor had *HER2* gene amplification, the patient could be enrolled in the trial (4). Another important aspect to mention, is the criteria for HER2 IHC tumor cell membrane staining. When FDA approved trastuzumab and HercepTest™, they also approved the IHC scoring criteria for the assay, which required a strong complete membrane staining in more than 10% of the tumor cells in order to be classified as HER2 IHC3+, as shown in **Table 1** (17). This staining requirement has been used in all pivotal breast cancer trials with trastuzumab including the metastatic and adjuvant indications, and subsequently also in relation to other HER2 inhibitors, such as pertuzumab and ado-trastuzumab emtansine.

In order to improve the accuracy and to standardize HER2 testing, the ASCO and the College of American Pathologists (CAP) issued a clinical practice guideline for HER2 testing in breast cancer in 2007 (36). As part of the guideline, in order to improve the robustness of the HercepTest™ scoring, it was suggested to change the requirements for HER2 IHC3+ from a > 10% strong complete membrane staining of the tumor cells to > 30%. This change was highly questioned as no clinical outcome data was available to support the 30% requirement, as all pivotal clinical trials with trastuzumab had used the original FDA approved scoring criteria (37, 38). The use of the suggested ASCO/CAP scoring criteria would identify a slightly different patient population compared to the populations in the different clinical trials with trastuzumab (39). However, with the revision of the ASCO/CAP guideline in 2013 and 2018, the scoring criteria became similar to the original FDA approved criteria (40, 41).

## Gastric Cancer

*HER2* amplification and/or HER2 over-expression have been reported in malignancies other than breast cancer, such as ovarian, prostate, colorectal, pancreatic, lung, and gastric cancers (42). In gastric cancer, amplification of the *HER2* gene and overexpression of the HER2 protein was first described in 1986 (43, 44). A large number of studies subsequently confirmed these findings, and several have shown that HER2 overexpression most likely acts as a negative prognostic marker in patients with gastric cancer (42, 45). Based on the results from the ToGA trial, the HercepTest™ was approved by FDA in 2010 as a companion diagnostic for trastuzumab for treatment of HER2 positive patients with metastatic gastric or gastroesophageal junction adenocarcinoma, as shown in **Figure 1**.

### HercepTest™ for Gastric Cancer

**Figure 3:**
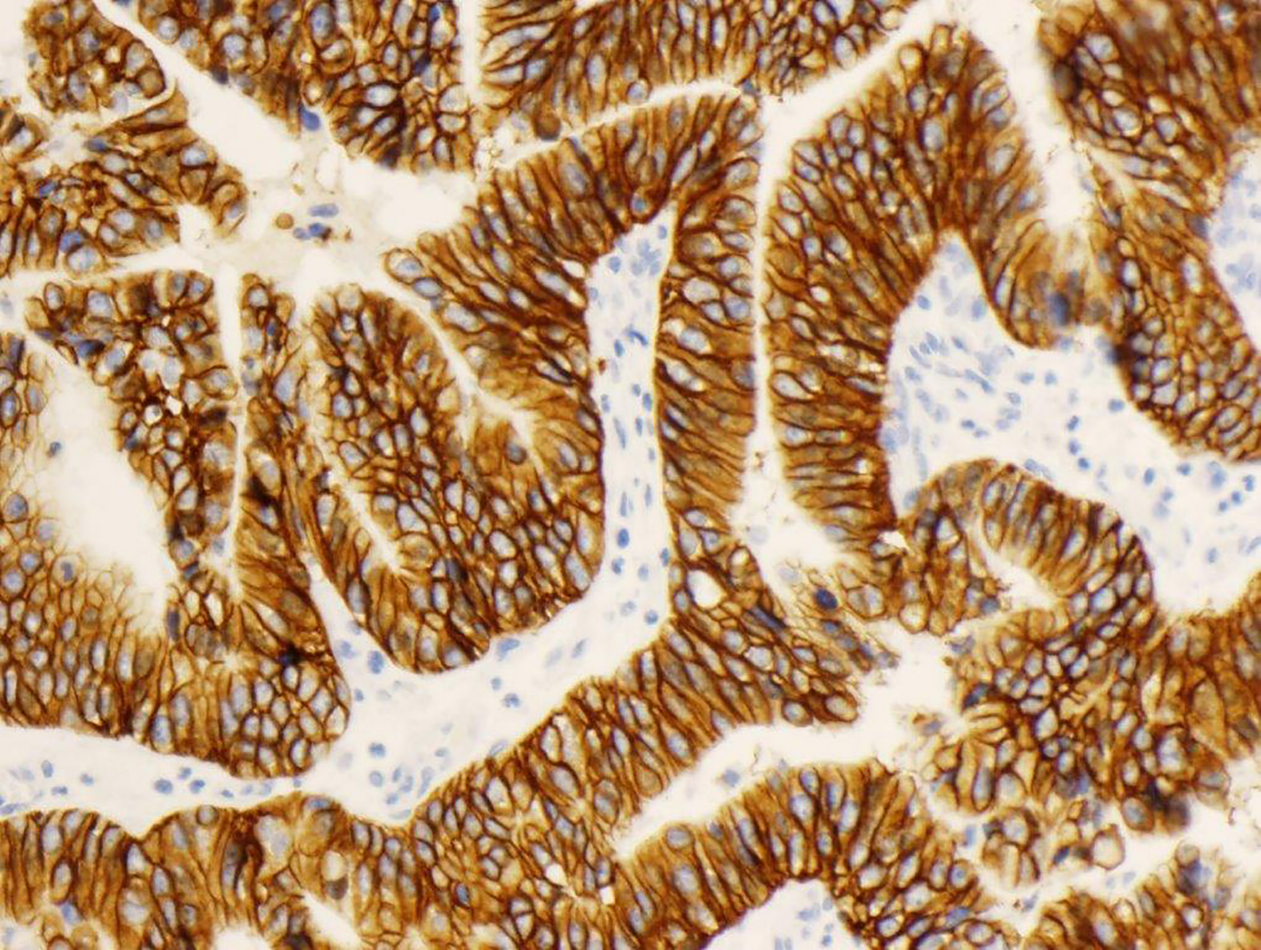
Adenocarcinoma of the stomach stained with HercepTest™, IHC3+ score.

Initially, when HercepTest™ was considered for testing of metastatic gastric tumor specimens, the interpretation and scoring criteria were based on the existing breast cancer criteria. However, due to marked differences in tumor biology between breast and gastric cancer tissue, modifications of the scoring criteria would be required before the assay could be used for enrollment of patients in clinical trials with trastuzumab. The modifications implemented in the scoring of gastric cancer specimens were based on input from a panel of international pathology and oncology experts, who recommended certain changes to the HercepTest™ scoring criteria used in breast cancer. The major difference between the interpretation and scoring criteria for the two indications was that specific criteria for biopsies were added for gastric cancer (46, 47). These changes were necessary to assess the tumor heterogeneity and incomplete HER2 cell membrane staining observed in gastric cancer tissues. The modifications agreed upon by the panel were incorporated in the scoring criteria for HercepTest™ as shown in **Table 2** (17). Due to the differences between gastric cancer and breast cancer tissues, the panel also recommended that both IHC and FISH testing should be performed for the enrollment of gastric cancer patients in clinical trials with trastuzumab (48). **Figure 3** shows a tissue section of an adenocarcinoma of the stomach with a strong expression of HER2 (IHC3+) following staining with HercepTest™.

**Table 2:** The FDA approved scoring criteria for HercepTest™ in gastric or gastroesophageal junction adenocarcinoma (17).

Score	HER2 Overexpression Assessment	Surgical Specimen – Staining Pattern	Biopsy Specimen – Staining Pattern
0	Negative	No reactivity or membranous reactivity in < 10% of tumor cells	No reactivity or no membranous reactivity in any (or < 5 clustered) tumor cell
1+	Negative	Faint/barely perceptible membranous reactivity in ≥ 10% of tumor cells; cells are reactive only in part of their membrane	Tumor cell cluster (> 5 cells) with a faint/barely perceptible membranous reactivity irrespective of percentage of tumor cells stained
2+	Equivocal	Weak to moderate complete, basolateral or lateral membranous reactivity in ≥ 10% of tumor cells	Tumor cell cluster (> 5 cells) with a weak to moderate complete, basolateral or lateral membranous reactivity irrespective of percentage of tumor cells stained
3+	Positive	Strong complete, basolateral or lateral membranous reactivity in ≥ 10% of tumor cells	Tumor cell cluster (> 5 cells) with a strong complete, basolateral or lateral membranous reactivity irrespective of percentage of tumor cells stained

### The ToGA Trial

Positive results from preclinical studies with trastuzumab in different HER2 *in vitro* and *in vivo* gastric cancer models as well as data from a few positive case studies lead to the initiation of the ToGA trial (42). The trial was designed as an open labeled, randomized multicenter phase III study in HER2-positive patients with histologically confirmed inoperable locally advanced, recurrent, or metastatic gastroesophageal junction adenocarcinoma (46). HER2 positivity was defined as either IHC3+ or positivity by *HER2* FISH (*HER2*/CEN17 ≥ 2), using HercepTest™ and the *HER2* FISH pharmDx assays, respectively. Based on the recommendations from the expert panel, both IHC and FISH tests were performed for almost all patients. After inclusion in the study, patients were randomized to receive chemotherapy (5-FU or capecitabine plus cisplatin) or chemotherapy plus trastuzumab. More than 3800 patients were screened for the study and 584 of these were enrolled in the ToGA trial. The primary endpoint was OS, with different secondary endpoints including ORR (46, 48). Overall, the ToGA trial showed that the combination of chemotherapy plus trastuzumab was superior to chemotherapy alone. The median OS increased from 11.1 to 13.8 months (HR 0.74; 95% CI, 0.60-0.91). For the group of patients who had received the combined treatment with chemotherapy and trastuzumab ORR was 47% and for the chemotherapy group 35%. A pre-planned exploratory analysis that looked at the efficacy in the different HER2 IHC scoring categories showed that the survival benefit provided by trastuzumab was dependent on the level of HER2 protein overexpression. The single subgroup of patients with the greatest survival benefit was the one with HER2 IHC3+. Here, the median OS increased to 17.9 months when trastuzumab was added to chemotherapy compared to chemotherapy alone with an OS of 12.3 months (HR 0.58; 95% CI, 0.41-0.81) (46, 48).

Based on the exploratory subgroup analyses from the ToGA trial, a specific HER2 testing algorithm was developed where the primary test was IHC with FISH reflex testing of HER2 IHC2+ patients. As more than 90% (533/584) of the patients enrolled in the ToGA trial had tumors with *HER2* gene amplification, it was argued that the selection criteria for treatment with trastuzumab should be both IHC3+ and gene amplification. In relation to the approval of trastuzumab for treatment of gastric cancer, this was in fact the position taken by the FDA, who recommended that reflex testing with FISH should be done for both IHC2+ and IHC3+ (17, 48). Furthermore, in the indication for use statement for HercepTest™, it says that the ToGA trial demonstrated that *HER2* amplification and protein overexpression are not as correlated as for breast cancer, thus a single method should not be used to determine HER2 status in gastric cancer patients (17). Looking specifically at the test results for the patients enrolled in the ToGA trial, the agreement between *HER2* amplification and HER2 overexpression is somewhat lower in gastric cancer than typically observed in breast cancer (49, 50). A relatively high number of *HER2* FISH positive patients were found among the HER2 IHC0 and IHC1+ tumors.

In 2010, the gastric cancer indication for trastuzumab was approved by the FDA together with HercepTest™ and the *HER2* FISH pharmDx assays. As shown in **Table 3**, these two assays are still the only available FDA approved companion diagnostics for this indication today. Similar to breast cancer, ASCO, CAP and the American Society for Clinical Pathology (ASCP) issued a practice guideline for HER2 testing in gastroesophageal adenocarcinoma in 2017 (52). As for the original ASCO/CAP breast cancer guideline, the ASCO/CAP/ASCP guideline for gastroesophageal adenocarcinoma deviates slightly from the original FDA approved scoring criteria. Also, here, the primary test is IHC, but FISH reflex testing should only be performed in HER2 IHC2+ patients, which is in contrast to the original FDA approved scoring criteria where a HER2 IHC3+ test result must also be confirmed by FISH testing.

**Table 3:** FDA approved companion diagnostics assays for HER2 targeted drugs. Not all the listed assays are currently available from the diagnostic manufactures (51).

Assay	Manufacturer	Breast cancer	Gastric cancer
***IHC Assays***			
Bond Oracle HER2 IHC System	Leica Biosystems	Trastuzumab	
HercepTest™	Agilent Technologies/Dako Denmark	Trastuzumab	Trastuzumab
		Pertuzumab	
		Ado-trastuzumab emtansine	
InSite™ Her-2/neu KIT	Biogenex Laboratories	Trastuzumab	
PATHWAY anti-Her2/neu	Ventana Medical Systems	Trastuzumab	
		Ado-trastuzumab emtansine	
***ISH Assays***			
*HER2* FISH pharmDx Kit	Agilent Technologies/Dako Denmark	Trastuzumab	Trastuzumab
		Pertuzumab	
		Ado-trastuzumab emtansine	
*HER2* CISH pharmDx Kit	Agilent Technologies/Dako Denmark	Trastuzumab	
INFORM™ *HER-2*/neu	Ventana Medical Systems	Trastuzumab	
INFORM™ *HER2* Dual ISH DNA Probe Cocktail	Ventana Medical Systems	Trastuzumab	
		Ado-trastuzumab emtansine	
PathVysion^®^ *HER-2* DNA Probe Kit	Abbott Molecular	Trastuzumab	
SPOT-LIGHT^®^ *HER2* CISH Kit	Life Technologies Corporation	Trastuzumab	
VENTANA *HER2* Dual ISH DNA Probe Cocktail	Ventana Medical Systems	Trastuzumab	
***NGS Assays***			
FoundationOne^®^ CDx	Foundation Medicine	Trastuzumab	
		Pertuzumab	
		Ado-trastuzumab emtansine	

IHC, Immunohistochemical; ISH, in situ hybridization; NGS, next generation sequencing.

## Other HER2 Assays

Shortly after the approval of HercepTest™ in 1998, the first *HER2* FISH assay that obtained FDA approval was the PathVysion^®^ *HER-2* DNA Probe Kit (Abbott Molecular). However, the indication was different compared to the HercepTest™, as the assay was not intended for use as a companion diagnostic for trastuzumab. According to the FDA Approval Order Statement, the results from the PathVysion^®^ Kit were intended as an adjunct to existing clinical and pathologic information used as prognostic factors in stage II, node-positive breast cancer patients. Furthermore, the statement said that the assay was indicated as an aid in predicting disease-free and overall survival in patients with stage II, node positive breast cancer treated with adjuvant cyclophosphamide, doxorubicin, and 5-fluorouracil (53). In 2001, the PathVysion^®^ Kit was finally approved as a companion diagnostic for trastuzumab (53, 54). Both the PathVysion^®^ *HER-2* DNA Probe Kit and the HercepTest™ became comparator assays for subsequently developed ISH and IHC assays, respectively. The subsequent FDA-approved ISH assays introduced the dual-color chromogenic visualization that enabled *HER2/CEN17* ratio determination by brightfield microscopy (**Table 3**) with excellent agreement to the comparator assays for HER2 status in breast cancer (55, 56).

Since 1998, the number of HER2 assays has steadily increased, and by the end of 2020, the total number of assays that have been approved by the FDA has reached 12, as shown in **Table 3** (51). However, not all the approved assays listed in the table are currently available from the diagnostic manufactures. The most recent approval of an assay for testing of *HER2* amplification was in 2017, when the next generation sequencing assay FoundationOne CDx™ (Foundation Medicine) was approved by the FDA as a companion diagnostic for trastuzumab, pertuzumab, and ado-trastuzumab emtansine in breast cancer (57). With respect to the validation of the FoundationOne CDx™ for testing *HER2* amplification, a concordance study to the *HER2* IQFISH pharmDx assay (Dako) was performed. Compared to the other HER2 assays listed in **Table 3**, the FoundationOne CDx™ is a laboratory developed test (LTD), which means it is a single-site assay which can only be performed at the site of Foundation Medicine.

In 2020, a second generation HercepTest™ for use in breast cancer was CE-IVD marked and made available in the European Union. CE-IVD marking means that the assay has been certified according to the Requirements of European Directive 98/79/EC of the European Parliament on *in vitro* diagnostic medical devices. The new assay, HercepTest™ mAb pharmDx (Dako Omnis), is intended for automated use on the Dako Omnis staining platform and is based on the primary monoclonal rabbit antibody, clone DG44 (58). In relation to the validation of the second generation HercepTest™, concordance studies were performed with both the first generation HercepTest™ and the *HER2* IQFISH pharmDx assay. The comparison to the first generation HercepTest™ was performed on 458 breast cancer specimens and the overall percent agreement (OPA) was 94.5% (95% CI, 92.1-96.3). The negative percent agreement (NPA) and positive percent agreement (PPA) for the comparison were 98.3% (95% CI, 95.7-99.3) and 93.3% (95% CI, 89.2-95.9), respectively. The comparison to *HER2* IQFISH pharmDx was performed on 422 breast cancer specimens and here, the results for NPA and PPA were 98.2% (95% CI, 95.4-99.3) and 93.1% (95% CI, 87.0-96.5), respectively (58).

## Conclusion

HercepTest™ has played a significant role in the development of HER2 targeted therapy and the treatment of thousands of patients with breast and gastric cancer. Without an accurate, robust and reliable assay for patient selection, most pharmacological targeted cancer therapies lose their value. In an interview with *Expert Review of Molecular Diagnostics* in 2015, Daniel Hays said that people need to value biomarker tests as much as they value drugs and that researchers should do biomarker studies with the same amount of rigor as therapeutic trials. He continued in the same interview by saying that: ‘A bad tumor biomarker test is as bad as a bad drug’ (59). However, we would like to take on a more positive attitude and turn the statement of Daniel Hayes around by saying: ‘A good tumor biomarker test is as good as a good drug’. For trastuzumab, the timely development of the CTA was of key importance. Without this predictive assay to enrich for those patients most likely to respond, the phase III trial in metastatic breast cancer, which lead to the approval of trastuzumab in 1998, would likely have failed. For the past two decades, HercepTest™ has played an important role in expanding the indication for trastuzumab as well as in the introduction of new HER2 targeted therapies. Trastuzumab and HercepTest™ have undoubtedly made an impact on the way we have perceived personal medicine for the past 20 years. Furthermore, it is important to emphasize that HercepTest™ was the first companion diagnostic ever approved by the FDA and more than 20 years of usage has shown its clinical impact.

## Author Contributions

JTJ, HW, JA, LA, DO, and JM contributed to the writing of this review. All authors contributed to the article and approved the submitted version.

## Funding

This article was funded by a grant from the Dx-Rx Institute, Fredensborg, Denmark.

## Conflict of Interest

The authors have all been involved in the development of either first and/or second generation HercepTest™. JTJ is a former employee of Dako and has worked as a consultant for Agilent Technologies, Euro Diagnostica, Oncology Venture, Azanta, Alligator Biosciences, and Leo Pharma and has given lectures at meetings sponsored by AstraZeneca, Merck Sharp & Dohme, and Roche. JTJ is employed by Dx-Rx Institute. HW is a former employee of Dako and currently an employee of Biovica International AB. JA is a former employee of Dako and Genentech and has worked as a consult for Medical Prognosis Institute, Oncology Venture, and Inbiomotion SL. LA, DO, and JM are employees of Agilent Technologies Denmark ApS, previously Dako, and are shareholders of Agilent Technologies Inc.

The authors declare that this article received funding from Dx-Rx Institute. The funder had the following involvement with the article: JTJ is an employee of the Dx-Rx Institute that paid the publication fee.

## Publisher’s Note

All claims expressed in this article are solely those of the authors and do not necessarily represent those of their affiliated organizations, or those of the publisher, the editors and the reviewers. Any product that may be evaluated in this article, or claim that may be made by its manufacturer, is not guaranteed or endorsed by the publisher.
